# Functional brain mapping using specific sensory-circuit stimulation and a theoretical graph network analysis in mice with neuropathic allodynia

**DOI:** 10.1038/srep37802

**Published:** 2016-11-29

**Authors:** Yuji Komaki, Keigo Hikishima, Shinsuke Shibata, Tsunehiko Konomi, Fumiko Seki, Masayuki Yamada, Naoyuki Miyasaka, Kanehiro Fujiyoshi, Hirotaka J. Okano, Masaya Nakamura, Hideyuki Okano

**Affiliations:** 1 Department of Physiology, Keio University School of Medicine, Shinjuku-ku, Tokyo 160-8582, Japan; 2 Imaging Analysis Laboratory, Laboratory Animal Research Department, Central Institute for Experimental Animals, Kawasaki-shi, Kanagawa 210-0821, Japan; 3 Department of Orthopaedic Surgery, Keio University School of Medicine, Shinjuku-ku, Tokyo 160-8582, Japan; 4 Department of Orthopedic Surgery, National Hospital Organization Murayama Medical Center, Musashimurayama-shi, Tokyo 208-0011, Japan; 5 Faculty of Radiological Technology, School of Health Sciences, Fujita Health University, Toyoake-shi, Aichi 470-1192, Japan; 6 Department of Pediatrics, Perinatal and Maternal Medicine, Graduate School, Tokyo Medical and Dental University, Bunkyo-ku Tokyo 113-8519, Japan; 7 Division of Regenerative Medicine, The Jikei University School of Medicine, Minato-ku, Tokyo 105-8461, Japan; 8 Laboratory for Marmoset Neural Architecture, Brain Science Institute RIKEN, 2-1 Hirosawa, Wako, Saitama 351-0198, Japan

## Abstract

Allodynia, a form of neuropathic pain, is defined as pain in response to a non-nociceptive stimulus. The brain regions responsible for pain, which are not normally activated, can be activated in allodynic mice by providing a suitable stimulus to Aβ-fibers, which transmit signals from tactile sensory fibers. Functional MRI (fMRI) can be used to objectively observe abnormal brain activation. In the present study, fMRI was conducted to investigate allodynia in mice; allodynia was generated by surgical injury at the L4 spinal nerve root, thus selectively stimulating sensory nerve fibers. In intact mice, only the primary somatosensory cortex (S1) was activated by stimulation of Aβ-fibers. Meanwhile, allodynic mice showed significantly higher BOLD signals in the anterior cingulate area (ACA) and thalamus. Using resting state fMRI, both degree and eigenvector centrality were significantly decreased in the contralateral S1, clustering coefficient and local efficiency were significantly increased in the ACA, and betweenness centrality was significantly higher in the ventral posterolateral nucleus of the thalamus. These results suggest that the observed abnormal BOLD activation is associated with defects in Aβ-fibers when Aβ-fibers in allodynic mice are selectively stimulated. The objective approach enabled by fMRI can improve our understanding of pathophysiological mechanisms and therapeutic efficacy.

Neuropathic pain is typically caused by traumatic injury of the nervous system, such as the spinal cord or peripheral nerves^1^. Neuropathic pain has historically been evaluated by behavioral analyses, including the von Frey filament test, Hargreaves test, and electrical stimulation-induced paw withdrawal (EPW) test^2^,^3^,^4^,^5^. These subjective measures of neuropathic pain can be influenced by several factors that involve both the subject and the observer. For instance, subjects tend to exhibit reduced pain sensitivity when a placebo is provided^6^. There is also no linear correlation between the stimulus strength and pain sensitivity resulting from nociception^7^. Even when a consistent nociceptive stimulus is applied, either through experience or mistaken assumption, sensitivity to pain is inconsistent^8^. One reason for this inconsistency is that neural activities are influenced by various factors, including peripheral or central sensitization, genetics, cognition, and emotion during the transmission of nociceptive stimuli to pain-receptive areas of the brain^9^. Another factor that influences the evaluation of neuropathic pain in behavioral analyses is observer bias. To overcome these issues, MRI may enable the objective and quantitative evaluation of pain^10^,^11^.

Previous studies have used intensity-based stimuli to investigate neuropathic pain in rodents, but these have not focused on the properties of peripheral nerve fibers^10^,^11^. Peripheral nerve fibers can be categorized according to their axonal diameter, degree of myelination, and transmission latency. Sensory fibers connected to the spinal dorsal horn are classified broadly into three types: Aβ-, Aδ-, and C-fibers. Aδ- and C-fibers mainly transmit nociceptive stimuli, whereas Aβ-fibers transmit tactile sensations. Individual sensory fibers (Aβ-, Aδ-, C-fibers) receive electrical stimuli of different frequencies (2000, 250, 5 Hz) that selectively reflect the physiological features of each fiber^2^,^12^. In the present study, to consider the properties of these different fiber types, peripheral fibers (Aβ-, Aδ-, C-fibers) were selectively stimulated to clarify their location of brain function, which enabled us to examine neuropathic pain in detail.

Allodynia, a type of neuropathic pain, is defined as pain in response to a non-nociceptive stimulus^13^. Aβ-fibers normally transmit tactile stimuli to the mechanoreceptive sensory area of the spinal cord. However, in allodynia, Aβ-fibers connect abnormally to the pain-transmission pathway, which may cause the symptoms associated with this disorder^14^,^15^,^16^. To evaluate neuropathic pain, Aβ-fibers must be selectively activated using a 2000 Hz stimulus of less than 2.2 mA^2^,^12^.

Recent research in brain imaging involving the integration of resting state functional connectivity MRI (rs-fc MRI) and graph theory has revealed some fundamental aspects of brain-network organization in neurological disorders. rs-fc MRI is a novel approach that examines spontaneous brain function by using blood oxygen level-dependent contrast in the absence of a task^17^. Brain connectivity in a mouse model of pain is not yet fully clear. In this study, fMRI scans were conducted using a mouse model of allodynia, in which allodynia was induced by peripheral nerve injury with a 2000 Hz stimulus. With the use of rs-fc MRI, we evaluated the properties of brain networks in activation areas during task fMRI.

## Results

**Figure 1:**
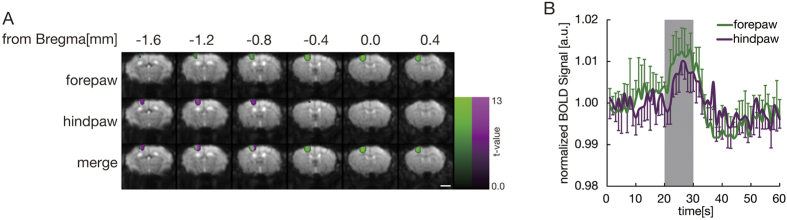
Segregation of BOLD signals detected after forepaw and hindpaw stimulation. BOLD signals were detected in regions adjacent to the contralateral S1 cortex of adult mice following stimulation of the forepaw (cyan) and hindpaw (magenta). The brain regions activated by 2000 Hz electrical stimulation of Aβ fibers were clearly visualized on BOLD echo planar images. *P* < *0.05* voxel-wise FWE corrected; Scale bar, 2 mm. (**B**) The increase in MR signal in the contralateral S1 cortex was closely correlated with the stimulus performed between 20–30 s (gray). X axis, scan time [sec]; Y axis, signal intensity; Error bar, SE; Strength of stimulation, 500 μA at 2000 Hz.

To precisely identify specific regions of the brain involved in nociception, a stereotaxic template of the wild-type mouse brain was created, and activation areas that responded to multiple stimulations were mapped onto the template. Electrical stimulation at 2000 Hz, equivalent to touch stimulation, was applied to both the left forepaw and hindpaw. On fMRI scans, the activated areas were clearly separated from each other, with the lateral side of the S1 region activated by forepaw stimulation and the medial side activated by hindpaw stimulation (Fig. 1A). The most activated voxel had a T value of 13.13 for forepaw stimulation and a T value of 10.48 for hindpaw stimulation. Stimulus-induced time-dependent changes in MR signal in the S1 area (Fig. 1B) revealed a close correlation between the timing of stimulation (gray) and increased BOLD signal intensity.

### Mapping the BOLD area from several sensory inputs

**Figure 2:**
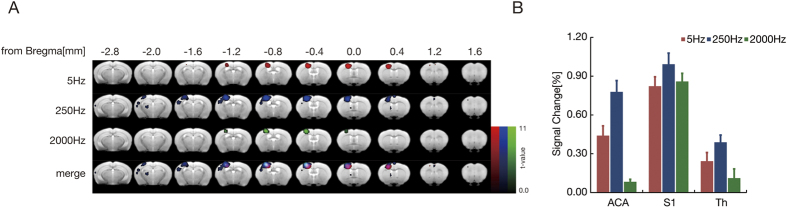
BOLD mapping by the selective stimulation of intact peripheral nerves. (**A**) The BOLD signal (red) from 5 Hz stimulation of C-fibers (nociceptive and thermal sensations) was specifically located in the contralateral ACA and S1 forepaw region. After 250 Hz stimulation of Aδ-fibers (fast pain) and Aβ-fibers (touch sensation), the signal (blue) was observed in the contralateral ACA, S1, and thalamus. After 2,000 Hz stimulation of Aβ-fibers, the activated area (green) was detected only in the contralateral S1 (**B**). To obtain the signal change (%), the MR signal in the ACA, S1, and thalamus was analyzed quantitatively. The results indicated that a significantly higher BOLD signal was detected in the S1 region with all types of sensory input through C-, Aδ- and Aβ-fibers, whereas those in the ACA and thalamus were only observed after painful stimulation through C- and Aδ-fibers. Intensity of stimulation, 500 μA; forepaw, *P* < *0.05* voxel-wise FWE corrected.

To elucidate the activated regions of the brain receiving different sensory perceptions, fMRI scans were conducted with three different subtypes of peripheral sensory stimuli. The specific mapping of BOLD signals resulting from selective stimulation of the forepaw is visualized in Fig. 2A. The ACA and S1 forepaw regions were activated by 5 Hz stimulation of C-fibers (nociceptive and thermal sensations). Highly increased BOLD signals were observed in the ACA, S1, and thalamus after 250 Hz stimulation of Aδ-fibers (fast pain) and Aβ-fibers (touch sensation)^18^. By contrast, 2000 Hz stimulation of Aβ-fibers induced BOLD signals only in the S1 region.

In a quantitative evaluation, BOLD signals in the ACA, S1, and thalamus were compared to baseline signals obtained without any stimuli present (Fig. 2B). In the ACA, significantly higher BOLD signals were only detected with painful stimuli through C- and Aδ-fibers but not with touch stimuli through Aβ-fibers. By contrast, in the S1 region, significantly higher BOLD signals were observed with every type of stimuli (through C-, Aδ- and Aβ-fibers).

### Behavioral assessment of neuropathic pain

**Figure 3:**
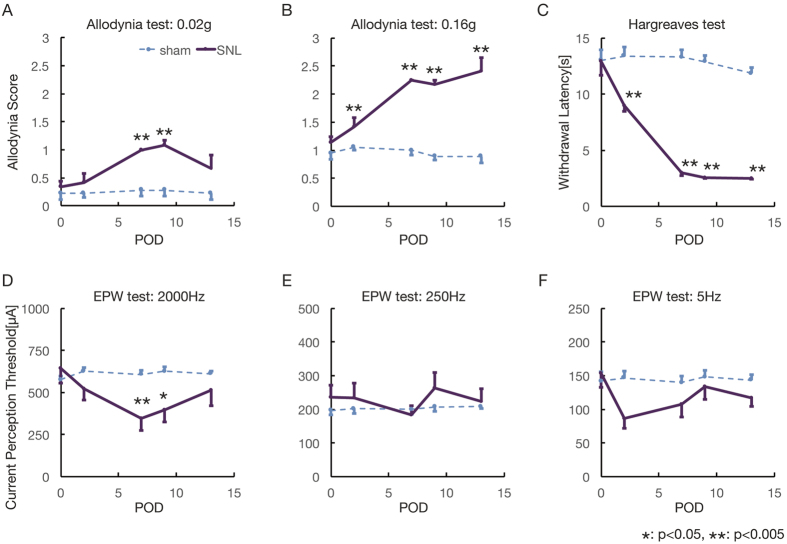
Behavioral assessment of pain and touch sensation in peripheral nerve injury model mice. (**A** and **B**) Mechanical allodynia was evaluated using von Frey filaments (0.02 g, 0.16 g). Seven days after peripheral nerve injury, significantly higher scores were detected for both 0.02 and 0.16 g stimulation. (**C**) Thermal allodynia was evaluated, and significant hypersensitivity was observed at postoperative day 2 and later. (**D–F**). The sensory fiber-selective threshold change was evaluated in the EPW test with electrical stimulation at 2000 Hz (**D**), 250 Hz (**E**), and 5 Hz (**F**). A significant difference in withdrawal threshold was detected at postoperative day 7 after 2000 Hz stimulation. Behavioral analyses indicated that mice at postoperative day 7 showed the greatest allodynia. 2-sample t-test (*p < 0.05, **p < *0.001*).

Next, a behavioral assessment was performed before surgery as well as 2, 7, 9, and 13 days after the operation in both the allodynic model and sham-operated mice. Mechanical allodynia was evaluated at each time point by stimulation with different nylon filaments (Fig. 3A,B). An abnormal threshold of sensation was observed 7 days after the operation with both the 0.02 g and 0.16 g filaments. The Hargreaves test (thermal paw-withdrawal test) was used to examine responses to thermal stimuli (Fig. 3C). Significantly greater sensitivity to heat was observed 2 days after the operation and thereafter. Next, the EPW test using different frequencies of electrical stimulation was performed to identify nerve fiber subtype-specific abnormalities. The withdrawal threshold under a 2000 Hz stimulus seven days after peripheral nerve injury was significantly lower than that pre-injury. These findings suggested that postoperative day 7 would be a suitable time point for MRI.

### Visualization of neuropathic pain

**Figure 4:**
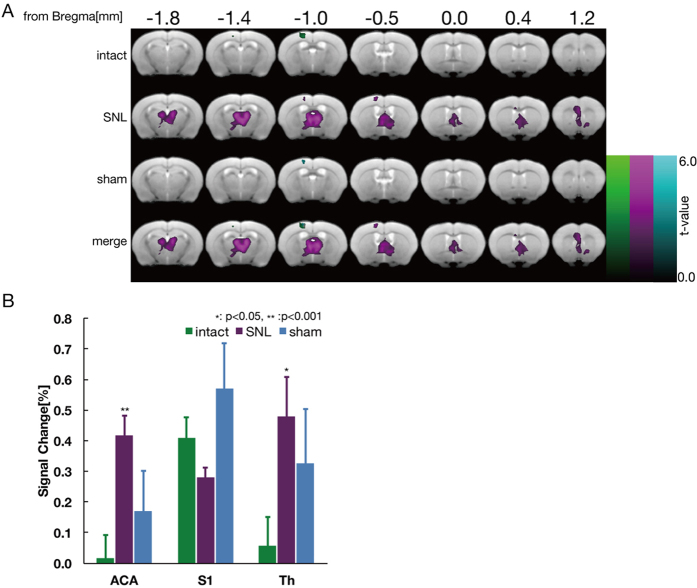
Visualization of allodynia in peripheral nerve injury model mice with fMRI. (**A**) BOLD signals in intact mice (green) were only observed in the contralateral S1 hindpaw region following 2000 Hz electrical stimulation. At postoperative day 7, an additional BOLD signal (magenta) was detected in the ACA, S1, and thalamus of the same animals. (**B**) For the quantitative assessment for allodynia, the signal change (%) was calculated by subtracting the MR signal in the ACA, S1, and thalamus, before and after peripheral nerve injury, from sham-operated mice. Significantly higher BOLD signals were observed in the ACA and thalamus after peripheral nerve injury. There were no significant differences between sham and SNL groups. Green, intact (preoperation); magenta, peripheral nerve injury; blue, sham-operated, MR signal threshold *P* < *0.05* voxel-wise FWE corrected, 2-sample t-test (*p < 0.05, **p < *0.005*).

As determined by the aforementioned fMRI observations (Fig. 2) and behavioral tests (Fig. 3), 2000 Hz electrical stimuli were applied to the mice seven days after peripheral nerve injury to visualize injury-induced allodynia. An averaged BOLD activation map from six mice before and after peripheral nerve injury is shown in Fig. 4A. Activation of Aβ-fibers with the application of 2000 Hz stimuli to the hindpaw of uninjured wild-type mice increased BOLD signal intensity in the contralateral S1 region in a pattern similar to that in Fig. 1A. Seven days after peripheral nerve injury, however, a highly increased BOLD signal was detected not only in the S1 but also in the ACA and thalamus. A quantitative evaluation of the BOLD signal, designated “signal change,” in the ACA, S1, and thalamus is shown in Fig. 4B. Stereotactic coordinates and statistical values are shown in Table 1. Significantly higher signal changes were observed in the ACA and thalamus of peripheral nerve injury model mice compared to pre-operative animals. This shift in signal change after injury in Fig. 4B parallels that in Fig. 2B. These results suggest that the nerve injury-induced activation of BOLD signal reflects the pain-receptive status from the stimulation of Aδ- and C-fibers.

### rs-fc MRI analysis

**Figure 5:**
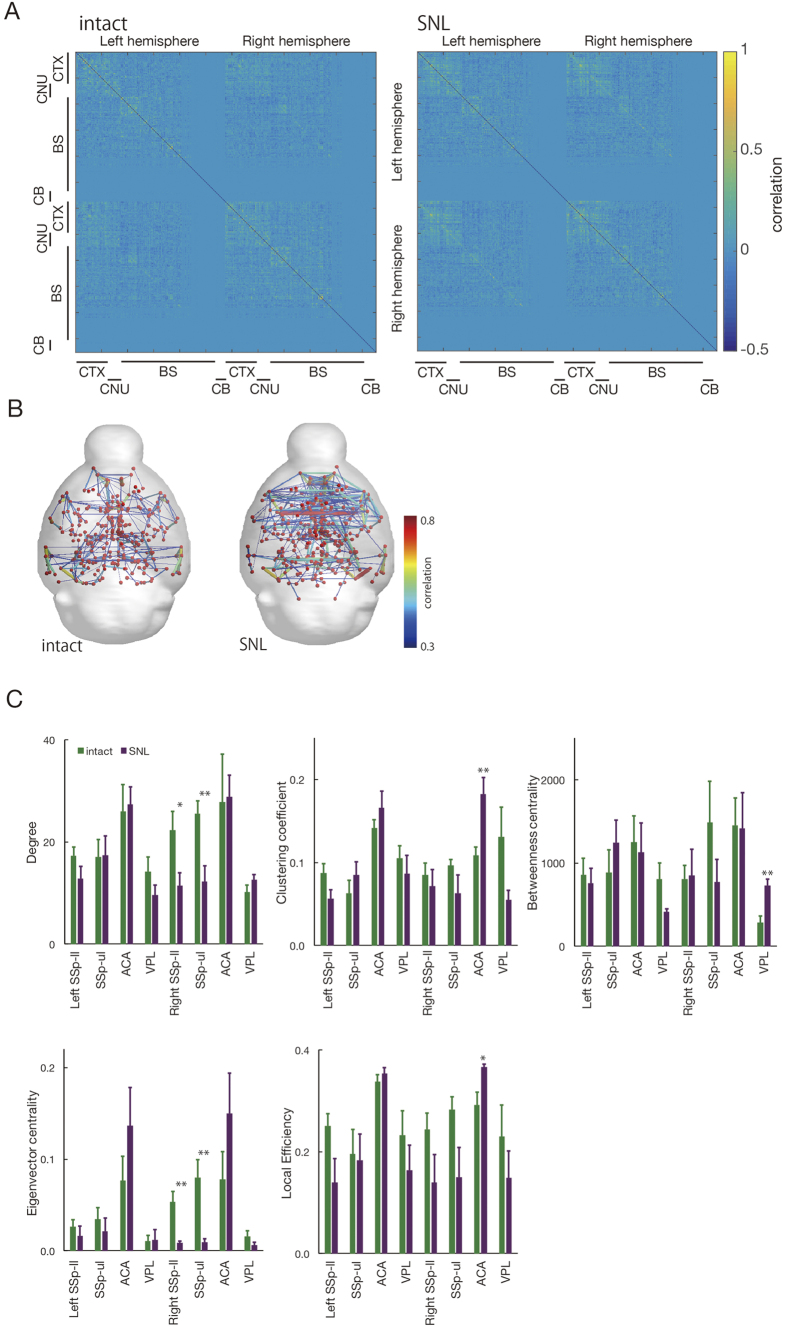
Visualization and graph theory analysis of brain networks in the peripheral nerve injury model mice with resting state functional connectivity MRI. (**A**) A connection matrix was expressed in terms of the temporal correlations between 576 architectonic subdivisions in the intact and spinal nerve ligation (SNL) mouse whole brain. Vertical and horizontal axes indicate the identification number of ROIs. The color bar shows the correlation between ROIs. (**B**) Brain networks were visualized in terms of 576 nodes and edges based on a connection matrix. (**C**) Characteristics of the brain network as calculated by a graph theory approach. Green, intact (preoperation); magenta, peripheral nerve injury. Paired *t*-test (*p < 0.05, **p < *0.001*).

To evaluate the interaction between activation areas in task fMRI, six mice were examined by rs-fc MRI before and after peripheral nerve injury. A connection matrix was expressed as the temporal correlation between architectonic subdivisions of each whole brain region (Fig. 5A), and brain networks, in which nodes represent ROI positions, and edges represent the correlations between nodes (Fig. 5B). The characteristics of the brain network were calculated using a graph theory approach (Fig. 5C). Degree and eigenvector centrality showed a significant reduction in the contralateral primary somatosensory area of lower limbs (SSp-ll, as defined in the Allen brain atlas) and upper limbs (SSp-ul) of peripheral nerve injury model mice compared with those in pre-operative animals. The clustering coefficient and local efficiency were significantly increased in the ACA. Significantly higher betweenness centrality was observed in the ventral postero-lateral nucleus of the thalamus (VPL). These results indicate that normal connection was decreased in S1, and the pain matrix that includes the ACA and VPL is complicated by neuropathic pain.

## Discussion

In this study, we developed an objective measure of allodynia using fMRI and an independent mouse model of neuropathic pain. Due to the small size of the mouse brain, it has been difficult to precisely evaluate responses to stimuli. One potential approach to solve this problem is the use of higher magnetic field MRI (9.4–11.7 T), as described in previous reports using mice^10^,^11^,^19^,^20^,^21^. However, it has been reported that when higher magnetic field strengths are used for fMRI, more signal deficits are detected in the brain due to distortion artifacts^20^. Another potential approach to overcome problems related to the small brain size is the use of a highly sensitive coil. In this study, we used a cryogenic RF probe with a 7 T MRI system. A significantly higher signal-to-noise ratio was achieved by cooling the electrical system of the RF coil to minimize noise^22^,^23^. These reports indicated that an approximately two-fold higher signal-to-noise ratio can be obtained with the use of a chilled RF coil compared to a non-cooled RF coil. In our study, we obtained images with high spatial resolution (0.2 × 0.2 × 0.5 mm) through the use of optimized scan parameters. To solve the problem of spatial distortion and signal loss due to susceptibility effects with the use of ultra-high field strength MRI, image acquisition is usually performed with segmented echo planar imaging (EPI)^24^. However, segmented EPI has two notable limitations. First, this method complicates the slice order and makes it difficult to correct gaps in slice timing. Second, the sampling interval increases in inverse proportion to the number of image segments. These artifacts increase in proportion to field strength; however, they can be reduced with the use of a system that combines 7T MRI and a cryogenic RF probe. Thus, high temporal resolution fMRI can be achieved with non-segmented EPI. By taking advantage of this system, the time course of changes in the intensity of BOLD signals could be investigated at a rate of one per second with single shot EPI. This increases the data reliability of the changes in the BOLD signal. In addition, high temporal resolution helps to estimate and filter out physiological noise, such as respiratory and heart rate signals^25^. These techniques also enable noise reduction for advanced fMRI techniques such as the analysis of functional connectivity^26^,^27^.

The choice of anesthesia is another area of concern with the use of fMRI in mice. In this study, we used medetomidine for longitudinal fMRI experiments. Typically, there are three major methods of anesthesia in murine fMRI: α-chloralose^21^, isoflurane^11^,^19^ and medetomidine^20^. Since isoflurane strongly suppresses neural activity and has a pronounced vasodilatory effect, it has not been frequently used in fMRI studies^20^. It has also been reported that a higher physiological correlation of the BOLD signal in a specific localization is observed with the use of α-chloralose and medetomidine for anesthesia compared with isoflurane^28^. α-Chloralose was previously used as a rodenticide, which clearly contraindicates the use of α-chloralose for long-term follow-up due to toxicity^29^,^30^. Medetomidine, which was used in this study, is one of the most suitable sedative agents for longitudinal studies because it provides a high correlation of BOLD signals in specific regions as well as minimal toxicity. Future studies may further investigate the effects of pain under various anesthetic conditions compared with the awake state.

Previous studies have identified a brain region termed the ‘pain matrix’ in humans, which includes the thalamus, the posterior and anterior insula, the secondary somatosensory cortex, the ACA, the periaqueductal gray matter, the central nucleus of the amygdala, and other regions^31^,^32^,^33^,^34^,^35^. In these regions, pain signaling pathways are classified into two major systems: a lateral nociceptive system based on the cortical projections of the lateral thalamic nuclei to S1 and S2 and a medial nociceptive system based on the medial thalamic group of nuclei with ACA projections^36^,^37^. The lateral nociceptive system mainly relays nociceptive information to somatosensory cortices. The medial nociceptive system is deeply involved in emotional aspects of pain. This thalamocortical system, which is mediated by medial thalamic nuclei, provides direct input into the ACA associated with directing attention, assigning response priorities, and conveying the ‘unpleasantness’ of pain^38^. One mouse fMRI study reported that a broadly activated region, which has an ambiguous edge but includes S1 and the thalamus, is observed following neural subtype non-specific electrical pain stimulation of peripheral nerve fibers^10^. One of the aims of the present study was to detect the pattern of BOLD signal activation under the selective stimulation of specific subtypes of sensory nerve fibers. The pattern of BOLD signal induced by subtype-selective stimulation (2000, 250, and 5 Hz) can be used to visualize projection patterns in a sensory fiber-specific manner (Aβ-, Aδ-, and C-fiber), as demonstrated in Fig. 2. Various factors contribute to the pathophysiology of allodynia: nerve injuries may contribute to re-organizing the connections between the nociceptors, mechanoreceptors and interneurons and the afferent neurons, which transmit pain information^39^,^40^. In the thalamus, the microglia may alter the properties of the secondary nociceptors^41^. Morever, the excessive recruitment of immune system cells such as monocytes/macrophages and T lymphocytes has been observed in the spinal cord^42^.

Figure 4 shows the BOLD pattern evoked by Aβ-fiber-selective stimulation in the ACA and thalamus in the allodynia model mice, which is similar to that induced by Aδ- and C-fiber stimulation in intact mice, indicating that abnormal BOLD activation in allodynia originates from defects in the Aβ fibers. Sensory subtype-specific fMRI allows us to perform precise evaluations in neurological disorders that include aberrant neural transmission, such as allodynia. In studies of patients with complex regional pain syndromes, it has been reported that allodynia led to widespread cerebral activations, including the contralateral S1 and motor cortex (M1), parietal association cortices (PA), bilateral S2, insula, frontal cortices, and both anterior and posterior parts of the cingulate cortex (aACC and pACC)^43^. Compared to these human studies, a similar pattern of up-regulated BOLD signal in allodynic mice was detected in the ACA but not the S1, as shown in Fig. 4. Decreased cerebral blood flow in the thalamus has also been reported in patients with post-traumatic neuropathic pain^44^. In the present study, allodynia-derived BOLD activation was detected in the thalamus of intact mice under tactile stimulation, originating from a decline from baseline of the blood flow. This result is similar to the case in humans with respect to the decrease of blood flow in the thalamus. These findings clearly suggest that the abnormal BOLD signal observed in the allodynia mouse model is nearly the same as the changes in blood flow in patients with allodynia. Thus, the fMRI analysis of pain as described in this study may be useful for evaluating neuropathic pain levels and for future drug screening in mice.

There was a significant difference between SNL and sham in the behavioral assessment and visualization of the BOLD activation map. However, in a detailed analysis of BOLD signal change, we found no significant difference. These results suggest that there may be an overall effect of sham surgery, which was not observed in the behavior test or BOLD activation map. A degree of pain may occur as a consequence of inflammation due to the removal of the transverse process at L5. In this surgical procedure model, phenotype assessments require careful attention to these subtle aspects.

fMRI studies have previously been used to identify brain regions that correspond to tasks. Recently, graph theory has been applied to the understanding of functional connectivity in MRI, particularly with regard to the resting state or endogenous fluctuations^45^,^46^. Comprehensive studies using resting state fMRI and a graph theory approach have shown that the statistical properties of endogenous fluctuations are modulated in Alzheimer’s disease^47^, Parkinson’s disease^48^, autism spectrum disorders^49^ and multiple sclerosis^50^. In this study, in the allodynia mouse model, the degree and eigenvector centrality were significantly decreased in the contralateral SSp-ll and SSp-ul, the clustering coefficient and local efficiency were significantly increased in the ACA, and the betweenness centrality was significantly higher in the VPL (Fig. 5). The degree reflects the number of significant functional connections of a node^48^, eigenvector centrality is a self-referential measure of centrality^51^,^52^, betweenness centrality is the fraction of all of the shortest paths in the network that contain a given node, the clustering coefficient is the fraction of triangles around a node, and the local efficiency is the global efficiency computed in the neighborhood of the node. These results indicate that the sensory information that comes from the neospinothalamic tract and the medial lemniscus of the posterior column-medial lemniscus pathway to become somatosensory through the VPL is reduced, and a pain matrix that includes the ACA and VPL may form a complicated circuit network. Future studies will be needed to clarify the direction of the transfer and to validate in detail the indicators of graph theory.

Here, we showed that integration of task fMRI with selective stimulation and rs-fc MRI with a graph theory approach can provide an objective measure of neuropathic pain. This approach can be used to improve our understanding of pathophysiological mechanisms and improve therapeutic efficacy.

## Methods

### Animals

Twelve intact adult mice (C57BL/6, male, approximately 10 weeks old; CLEA Japan Inc., Tokyo, Japan) were used in this study: six adult mice were used for the peripheral nerve injury model and six adult mice for the sham-operated model. Mice were housed in groups under a 12-hour light/dark cycle with access to food and water *ad libitum*. Room temperature was set at a consistent 23 ± 1 °C. All animal experimental procedures were performed in accordance with the Laboratory Animal Welfare Act and the Guide for the Care and Use of Laboratory Animals (National Institutes of Health, Bethesda, MD, USA). All experiments were approved by the Animal Study Committee of the Central Institute for Experimental Animals (approval number: 12014).

### Allodynia mouse model

To prepare mouse models of allodynia, peripheral nerve injury at the distal side of the dorsal root ganglion (DRG) was induced in six wild-type adult mice, as previously described^53^,^54^. In brief, after mice were deeply anesthetized with 2–3% isoflurane (Foren; Abbott, Tokyo, Japan), a midline incision was made in the dorsal region of L6 to L4 and the transverse process at L5 was removed using forceps. The exposed L4 spinal nerve root was compressed with forceps and the distal region was immediately transected from the site of compression. The muscle layer and skin were closed with sutures. In the sham-operated group, the L4 spinal nerve root was exposed without any injury. All animals recovered under observation in a heated cage.

### Behavior analyses

To evaluate allodynia, the von Frey filament test, Hargreaves test, and EPW test were performed 0, 2, 7, 9, and 13 days after the operation. To reduce experimental variance, mice were habituated in each experimental environment for 30 minutes. Each behavioral test is described below:

1. von Frey filament test. Mechanical allodynia was examined by pressing two different filaments (0.02 g and 0.16 g) against the underside of the hindpaw. Allodynia was scored as follows: 0) no response; 1) slow and/or slight response to the stimulus; 2) quick withdrawal response away from the stimulus without flinching or licking; or 3) intense withdrawal response away from the stimulus with brisk flinching and/or licking^3^.
2. Hargreaves test. To evaluate thermal allodynia, flight reactions were examined during the application of heat to the underside of the hindpaw^4^,^5^. The cut-off time for this test was set at 20 s.
3. EPW test. To evaluate the paw withdrawal response using an EPW test, electrical stimuli with a frequency of 5, 250, and 2000 Hz were applied to the left hindpaw to activate C-, Aδ-, and Aβ-fibers. The intensities that corresponded to the observation of paw withdrawal were defined as threshold values^55^. The stimulation intensity was automatically adjusted from 0 to 3000 μA in increments of 10 μA for 2000 Hz, 25 μA for 250 Hz, and 100 μA for 5 Hz. Behavioral indicators were analyzed to determine the inter-subject standard error, and groups were compared with the application of a 2-sample t-test.

### MRI measurement

fMRI was performed using a 7.0 tesla MRI system equipped with actively shielded gradients at a maximum strength of 700 mT/m (Biospec; 70/16 Bruker BioSpin, Ettlingen, Germany) and with a cryogenic quadrature radio frequency (RF) surface probe (CryoProbe; Bruker BioSpin AG, Fällanden, Switzerland) to improve sensitivity^9^,^10^,^19^,^22^,^23^. The respiration and rectal temperature of the animals were monitored during measurements.

A B0 shimming method was used to improve image quality with field mapping (MAPSHIM, ParaVision 5). As a reference for brain anatomy, high-resolution T_2_-weighted images (T_2_WIs) of the whole brain were acquired using a Rapid Acquisition with Relaxation Enhancement (RARE) method with the following parameters: effective time to echo (TE), 48 ms; time to repetition (TR), 6100 ms; RARE factor, 8; number of averages, 4; spatial resolution, 75 × 75 × 300 (μm)^3^; and number of slices, 52. Blood oxygenation level-dependent (BOLD) fMRI was acquired by a gradient echo-echo planar imaging method with the following parameters: TE, 20 ms; TR, 1000 ms; flip angle, 55°; number of averages, 1; spatial resolution, 200 × 200 × 500 (μm)^3^; and number of slices, 16. These parameters were used in all of the experiments described below.

Thirty minutes before fMRI acquisition, the anesthetic was changed from isoflurane to medetomidine (Orion Pharma, Espoo, Finland) administered subcutaneously via a 0.3 mg/kg bolus and 0.6 mg/kg/h infusion^20^. The left forepaws of healthy mice were stimulated electrically (500 μA) at specific frequencies (5 Hz, 250 Hz, and 2000 Hz), and fMRI scanning was performed. A block-design stimulation paradigm was used and consisted of five blocks, where each block comprised a 10 s stimulation followed by a 50 s resting condition. For electrical stimulation, two needle-type electrodes (NM-715S, SS-403J, SEN-7203, Nihon Kohden, Tokyo, Japan) were inserted under the skin between the second and fourth digits of the left forepaw.

The second experiment focused on 2000 Hz electric stimulation. This stimulation selectively depolarizes Aβ fibers, which does not normally invoke pain. fMRI was performed in allodynia model mice before the operation and seven days after the operation. Two needle-type electrodes were inserted under the skin between the second and fourth digits of the left hindpaw.

rs-fc MRI was applied in the same allodynia model mice before the operation and seven days after the operation. The scan repetition was 600 times, and the other acquisition parameters were the same as those in task fMRI.

### Data analysis

The task fMRI data analysis was performed using SPM8 (Wellcome Trust Centre for Neuroimaging, UCL Institute of Neurology, London, UK) and tailored software in MATLAB, which included adjustments for the timing of slice acquisition, motion correction, and co-registration of different mouse brains to a stereotaxic MRI brain template for intact C57BL/6 mice. The normalized functional images were smoothed with a 0.6 mm full width at half maximum (FWHM) filter. Statistical t-maps were calculated using a generalized linear model (GLM) analysis regarding the stimulation paradigm, delayed vascular response and head movement. Activation was detected using a statistical threshold of *P* < 0.05 (corrected for the family-wise error rate using the random field method) for all experiments^56^,^57^. A region of interest (ROI) for the calculation of BOLD signal changes was defined as a sphere of radius 0.6 mm, centered on a highly activated voxel of each region of activation (Table 2). The BOLD signal change in the ROI was calculated as the average derived value with stimulation divided by the baseline MR signal. The BOLD signal change was analyzed to determine the inter-subject standard error, and groups were compared by applying a 2-sample t-test.

The rs-fc fMRI data analysis of the geometric transformation was the same as that for task fMRI, as previously described. A functional connectivity analysis was performed using CONN^58^. A temporal band-pass filter was applied in the 0.009 Hz to 0.1 Hz range^59^. The voxel-averaged signal was determined over the entire rs-fMRI time-course and regressed out of the data together with the motion correction parameters by SPM8^60^. Five hundred seventy-six predefined regions based on connectional and architectonic subdivisions in the mouse brain atlas were used by combining atlases provided by the Allen Institute^61^,^62^. A functional correlation matrix was calculated on the basis of temporal correlation of BOLD signal by ROI to ROI. The node coordinate was determined by calculating centroids of the volume of each region. The edge was expressed as the temporal correlation of BOLD signals between nodes. The brain networks were visualized with the BrainNet Viewer^63^ (http://www.nitrc.org/projects/bnv/).

A graph theory analysis based on an undirected, weighted connection matrix was performed with the Brain Connectivity Toolbox^52^. Local measures included degree, eigenvector centrality^51^, clustering coefficient^64^, local efficiency^65^ and betweenness centrality^66^. Graph theory indicators were analyzed to determine the inter-subject standard error, and groups were compared using a paired t-test.

## Additional Information

**How to cite this article**: Komaki, Y. *et al*. Functional brain mapping using specific sensory-circuit stimulation and a theoretical graph network analysis in mice with neuropathic allodynia. *Sci. Rep.* **6**, 37802; doi: 10.1038/srep37802 (2016).

**Publisher's note:** Springer Nature remains neutral with regard to jurisdictional claims in published maps and institutional affiliations.

## Figures and Tables

**Table 1:** Stereotactic coordinates and statistical values of the activation area in allodynia model mice by task fMRI.

	S1					ACA					Thalamus				
	x[mm]	y[mm]	z[mm]	t-value	p-value	x[mm]	y[mm]	z[mm]	t-value	p-value	x[mm]	y[mm]	z[mm]	t-value	p-value
**intact**	−1.2	0.6	−1.0	2.5	2.3 × 10^−8^										
**allodynia**	−1.0	0.6	−0.5	2.8	2.3 × 10^−10^	0.0	2.3	1.3	2.9	6.7 × 10^−17^	0.7	3.4	−1.1	5.0	1.6 × 10^−34^
**sham**	−1.2	0.4	−1.0	1.1	1.1 × 10^−6^										

Stereotactic coordinates are defined by the distance from the bregma with a standardized brain template; t- and p-values are the values for the points of the coordinate.

**Table 2:** ROI coordinates for the calculation of BOLD signal changes.

	S1			ACA			Thalamus		
	x[mm]	y[mm]	z[mm]	x[mm]	y[mm]	z[mm]	x[mm]	y[mm]	z[mm]
**intact**	−1.20	0.64	−0.96	0.00	2.32	1.28	0.72	3.36	−1.12
**allodynia**	−1.04	0.56	−0.48	0.00	2.32	1.28	0.72	3.36	−1.12
**sham**	−1.44	0.48	−0.32	0.00	2.32	1.28	0.72	3.36	−1.12

The ROI for the calculation of BOLD signal changes was defined as a sphere of radius 0.6 mm, centered on a highly activated voxel of each region of activation.
